# Metabolite Biomarkers Linking a High-Fiber Rye Intervention with Cardiometabolic Risk Factors: The RyeWeight Study

**DOI:** 10.1021/acs.jafc.5c01415

**Published:** 2025-08-21

**Authors:** Andrea Unión Caballero, Tomás Meroño, Sebastian Åberg, Elise Nordin, Johan Dicksved, Alex Sánchez-Pla, Marta Cubedo, Francisco Carmona-Pontaque, Kia No̷hr Iversen, Miriam Martínez-Huélamo, Anna Guadall, Rikard Landberg, Cristina Andrés-Lacueva

**Affiliations:** 1 Biomarkers and Nutrimetabolomics Laboratory, Department de Nutrició, Ciències de l’Alimentació i Gastronomia, Institut de Recerca en Nutrició i Seguretat Alimentària (INSA-UB), Facultat de Farmàcia i Ciències de l’Alimentació, 16724 Universitat de Barcelona, Barcelona 08028, Spain; 2 Centro de Investigación Biomédica en Red de Fragilidad y Envejecimiento Saludable (CIBERFES), Instituto de Salud Carlos III, Madrid 28029, Spain; 3 Department of Life Sciences, Division of Food and Nutrition Science, 11248 Chalmers University of Technology, Gothenburg SE-412 96, Sweden; 4 Department of Applied Animal Science and Welfare,Nutrition and Management, Uppsala SE-750 07, Sweden; 5 Department of Genetics, Microbiology and Statistics, University of Barcelona, Barcelona 08028, Spain

**Keywords:** metabolomics, biomarker, wholegrain rye, refined wheat, cardiometabolic health, gut microbiota, randomized controlled trial

## Abstract

Wholegrain rye, considered one of the cereals with the highest content of dietary fiber and bioactive compounds, has been linked with reduced risk of cardiometabolic diseases. Thus, biomarkers reflecting its intake and/or the metabolic effect after consumption are essential to better elucidate its health effects. Our aim was to identify plasma metabolite biomarkers associated with a high-fiber rye intervention and to assess the associations between these metabolites, gut microbiota composition, and cardiometabolic risk factors in a 12-week randomized controlled trial comparing a hypocaloric diet with high-fiber rye (*n* = 108) or refined wheat (*n* = 99) in participants with obesity. Rye intervention increased plasma concentrations of benzoxazinoids (DIBOA-S) and phenylacetamides (2-HPA-S and 2-HHPA-S), gut microbial metabolites (indolepropionic acid, 2-aminophenol, enterolactone sulfate, and enterolactone glucuronide), betainized compounds (pipecolic-betaine), phenolic acids (2,6-DHBA and gallic acid-4-sulfate), and diverse endogenous metabolites. Microbiota composition changes were increased *Eubacterium xylanophilum* and *Agathobacter* and decreased *Ruminococcus torques* and *Romboutsia*. Moreover, the intervention effect was mostly captured by changes in metabolites and gut microbiota compared to clinical variables. Gallic acid-4-sulfate and phenylacetamides were associated with reductions in weight, fat mass, BMI, or fasting insulin levels even after adjusting for plasma alkylresorcinols, used as markers for rye intake compliance. Altogether, these metabolites may constitute biomarkers of wholegrain rye cardiometabolic effects.

## Graphical abstract

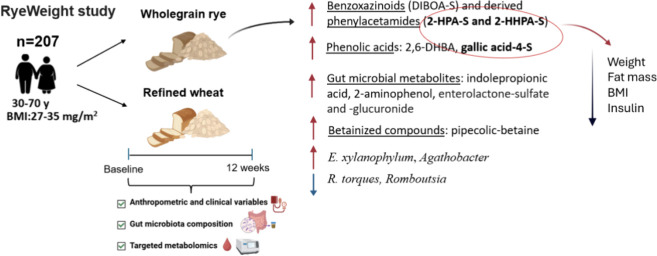

## Introduction

Wholegrain cereals constitute one of the main sources of dietary fiber in human diet, and their intake has been associated with a reduced risk of developing noncommunicable diseases, including cardiovascular diseases, obesity, type 2 diabetes, and some cancers.^1,2^ However, most of the cereals are consumed as refined grains, where the nutrient-rich bran and germ have been removed. The specific components and mechanisms through which wholegrain foods contribute to health are not fully characterized, but their high content in dietary fiber is well recognized as one of the important factors behind beneficial health effects. Dietary fiber found in wholegrains is typically partly fermented by the gut microbiota to produce fiber-specific metabolites that have been linked to beneficial health effects.^3–5^ In addition to dietary fiber, wholegrain foods contain numerous vitamins, minerals, phenolic compounds, and other phytochemicals, located in the bran or in the germ, all of which may additionally contribute to these protective effects.^5,6^

Wholegrain intake has been associated with improved cardiometabolic health.^7–10^ Specifically, a high wholegrain intake has shown an inverse association with body fat,^11^ blood lipids,^12^ systemic inflammation,^13^ and glycemia.^14^ In addition, the effects may differ between different types of cereals, which motivates assessment of their effects separately.^15^ Wholegrain rye is one of the cereals with the highest content of dietary fiber, and it contains a large variety of bioactive compounds.^5,16^ Phytochemicals found in rye include phenolic acids, lignans, alkylresorcinols, benzoxazinoids, and betaines,^5^ of which some have been proposed as biomarkers for rye intake or even mediators for its health benefits.^5^ Given its substantial content of bioactive compounds, rye has been suggested to be superior to other wholegrain cereals in terms of health promotion.^17^ Indeed, high-fiber wholegrain rye food has been shown to increase satiety compared to refined wheat products^18^ and has been linked to reductions in postprandial insulin,^19^ serum lipids, and the inflammatory marker C-reactive protein.^20^

To elucidate the effects of wholegrain rye intake, it is essential to have reliable and accurate estimators able to reflect the intake and/or the metabolic effect after its consumption. Moreover, a comprehensive assessment of potential molecular mediators of effects is of importance. Since the absorption of food components vary across individuals dependent on gut microbiota or genetic characteristics, measurements of the plasma concentration rather than the self-reported intake of specific components may be more related to health.^21^ Moreover, metabolite biomarkers may also reflect host and gut microbiota metabolic processes that are behind these associations with cardiometabolic risk factors.^22–25^ This could ultimately lead to a better understanding of the specific metabolic impact of wholegrain rye intake and guide future precision nutrition interventions aiming to maximize health effects of wholegrain rye intakes for improving cardiometabolic health.

The aims of this study were to identify plasma metabolite biomarkers increased by a high-fiber wholegrain rye intake and 2) to assess the associations between the changes in metabolites, gut microbiota composition, and cardiometabolic risk factors after a high-fiber rye intervention in a 12-week weight loss trial.

## Methods

### Study Design

A complete description of the study design and detailed procedures have previously been reported by Iversen et al.^26^ Briefly, the RyeWeight study was designed as a randomized controlled parallel study in overweight/obese participants, with the primary aim of investigating effects of hypocaloric diets with high-fiber wholegrain rye foods versus refined wheat foods on body weight and fat mass. After a 2-week run-in period, where all participants consumed wheat products, participants were randomized (1:1) to consume either rye products or wheat products as part of habitual diets for 12 weeks. During all 14 weeks, participants received dietary guidance from dieticians, aiming at a 500 kcal/day energy deficit to induce weight loss. Participants were instructed to avoid consumption of cereals not included in the study during all 14 weeks. At weeks 0, 6, and 12 of the parallel intervention phase, participants underwent a clinical examination, including anthropometric measurements, a dual-energy X-ray absorptiometry (DEXA) scan, and fasting blood and fecal sample collection.

### Ethical Considerations and Registration

The study was conducted in Uppsala, Sweden, between September 2016 and December 2018. All participants gave written consent after having received oral and written information about the study prior to initiating the screening procedure. The study was approved by the Ethical Review Board in Uppsala (Dnr: 2016/254) and registered at www.clinicaltrials.gov (Identifier: NCT03097237). The study was conducted in accordance with the Declaration of Helsinki.

### Study Population

Men and women aged 30–70 years, with a BMI of 27–35 kg/m^2^, were eligible to participate in the study. Detailed inclusion and exclusion criteria have been described elsewhere.^26^ Participants were required to lose ≥0.5 kg during the 2-week run-in period in order to be randomized into the 12-week parallel intervention phase. Participants who completed the 2-week run-in period were randomized 1:1 to receive either rye or wheat products for the 12-week parallel intervention phase. In total, 242 participants completed the run-in period with sufficient weight loss and were enrolled and randomized into the 12-week parallel intervention.

### Intervention Products

The intervention products consisted of breakfast cereals, crisp bread, and soft bread in both the rye group and the wheat group. Participants were instructed to consume a fixed amount of products per day (650 kcal/day, corresponding to approximately 30–50% of the participants daily energy intake) and record their intake in a precoded compliance journal. All intervention products were provided. Additionally, alkylresorcinols were measured in plasma as a supporting measure of compliance.^27^ The daily amount of rye products provided approximately 30 g of dietary fiber/day, whereas the wheat products provided 8 g of dietary fiber/day. In addition, participants were instructed not to consume any other cereals than the ones they received from the study, except for very small amounts of “hidden” cereal (e.g., thickening in sauces). Every day during the study, the participants filled in a precoded compliance journal where they ticked off the products they consumed.

### Clinical Examination and Biological Samples

At week 0, week 6 and week 12 participants attended an examination visit, where blood sampling, fecal sample collection, DEXA scan, and clinical and anthropometric examination were conducted after an overnight fast. Fasted blood samples were collected and were centrifuged, aliquoted, and stored at −80 °C until analysis. In addition, eating behavior was assessed at the first screening visit using the 21-item Three Factor Eating Questionnaire (TFEQ), which evaluates the participant’s behavior regarding three different domains: cognitive restrained eating, uncontrolled eating, and emotional eating.^28^

### Plasma Alkylresorcinol Levels

Plasma alkylresorcinols were measured at Chalmers Mass Spectrometry Infrastructure as previously reported by Iversen et al.^26^ Alkylresorcinol levels were analyzed in EDTA plasma using liquid chromatography tandem mass spectrometry, following a method reported elsewhere.^27^ The total plasma alkylresorcinol concentration was calculated as a sum of homologues C17:0–C25:0.

### Metabolomics Analysis of Plasma Samples

Plasma metabolomics analysis was performed in samples from baseline and 12-week time points at the Nutrimetabolomics lab of the University of Barcelona, following the targeted procedure described by González-Domínguez et al.^29^ Overall, this method developed in-house includes a large variety of phenolic acids, gut microbiota fermentation metabolites derived from polyphenols and amino acids, other food-related metabolites, and endogenous metabolites, such as carnitines, fatty acids, amino acids, and amino acid derivatives. Plasma samples were subjected to a protein precipitation procedure with minor modifications using a Sirocco Plate (Waters, Milford, Massachusetts, USA), as previously described.^29^ Briefly, 100 μL of plasma samples was spiked with 10 μL of 1 mg/L myristoyl-l-carnitine d9 and ferulic acid 13C3 in water. The samples were subsequently mixed with 500 μL of cold acetonitrile (−20 °C) containing 1.5 M formic acid and 10 mM ammonium formate in the plate, vortexed for 1 min, and kept at −20 °C for 10 min to promote protein precipitation. A Waters Positive Pressure-96 Processor was used to collect the extracts in 96-well collection plates, which were taken to dryness under a stream of nitrogen gas. Finally, the samples were reconstituted in 100 μL of water:acetonitrile (80:20, v/v) containing 0.1% formic acid (v/v) and 100 μg/L of the taxifolin and caffeine 13C3 as external standards. Clean extracts were then transferred to 96-well plates for further analysis.

Analyses were carried out by ultrahigh-performance liquid chromatography coupled to QTRAP spectrometry (UHPLC-QTRAP), using the operating conditions described elsewhere.^29^ Calibration curves were prepared at 10 concentration levels in the range 0.1–2000 μg/L. Compounds lacking the corresponding commercial standard were semiquantified using the calibration curves of structurally similar metabolites (see Table S1 for details). Sciex OS 2.1.6 software was used for data acquisition and processing.

### Metabolomics Data Preprocessing

Metabolomics data preprocessing was performed using the POMA R/Bioconductor package (https://github.com/nutrimetabolomics/POMA).^30^ Data preprocessing included the removal of metabolites with more than 40% missing values for endogenous metabolites and more than 80% missing values for those that were exogenous. The imputation of the remaining missing values was conducted using the KNN algorithm, and data were scaled and normalized using Pareto scaling and log transformation. The working metabolomics data set comprised 307 metabolites, including polyphenols and their metabolites derived from gut microbiota fermentation and/or host metabolism, and gut microbiota metabolites derived from dietary fiber components (*n* = 207, observations = 414).

### Faecal Microbiota

Participants arrived at the clinic after an overnight fast and brought a faecal sample. Stool samples were collected by the participants using EasySampler Faeces Collection Kit (GP Medical Devices Ltd., Holstebro, Denmark), containing a faecal collection tube (Sarstedt AG & Co., Munich, Germany). Stool samples were stored at −80 °C. DNA extraction and 16S rRNA gene amplicon sequencing procedure have been described in detail elsewhere.^31^ Total DNA was extracted from the fecal samples using a QIAamp Fast DNA Stool Mini Kit (Qiagen, Hilden, Germany) according to the protocol from the manufacturer, and amplicons from the V3 and V4 regions of the 16S rRNA gene were generated from the extracted DNA using the primers 341F and 805R. The amplicons were sequenced on the Illumina platform at Novogene (Beijing, China). The raw demultiplexed reads from the sequencing were processed using the DADA2 pipeline to denoise dereplicate reads, merge pair end reads, and remove chimeras.^32^ Amplicon sequence variants (ASVs) were assigned to reference sequences using the naive Bayesian classifier called with the assign Taxonomy command^33^ against the SILVA rRNA database.^34^ For the analysis, we used samples from baseline and 12-week time points, and we selected the genera with abundance counts greater than 100 in at least 5% of the participants. Robust centered log ratio (“rclr”) transformation with the “vegan” R-package was applied for the data.^35^

### Statistical Analyses

Mean and standard deviation or median (Q1–Q3) were used to describe variables following a Gaussian or skewed distribution, respectively. Mann–Whitney *U* test was used to assess differences in anthropometrics and metabolic characteristics at baseline between participants from both treatment arms. The intervention effect both on cardiometabolic risk factors and on plasma metabolome was assessed using linear mixed models including individual-specific random effects, and diet (rye vs wheat) and time (week 0 vs week 12) as main effects, with its two-way interaction. Random effects for the models were subject ID (random intercepts), since addition of random slopes did not improve the models’ likelihood ratio. *P*-values for treatment, for time, and for time × treatment interaction were obtained. *P*-values for interaction were adjusted for multiple comparisons using the Benjamini–Hochberg false discovery rate (FDR). FDR-adjusted *p*-values <0.05 were considered significant. Minus log10 of the FDR-adjusted *p*-values and log2 fold change of the median normalized metabolite levels were calculated and represented in a volcano plot. Post hoc comparisons among treatment-time groups were conducted using the “emmeans” R-package, obtaining the estimated marginal means for linear mixed models. Boxplots with the normalized concentration of the metabolites were represented.

Multivariate analyses were conducted using multilevel partial least-squares-discriminant analysis (PLS-DA) with the “mixOmics” R-package.^36^ Multilevel analysis properly deals with data that have a repeated design (prior- and post-treatment values in this case), increasing the quality of the analysis.^37^ Variable Importance in Projection (VIP) scores for diet discrimination in the PLS-DA model were additionally obtained and plotted.

To select features associated with the dietary intervention, we used a machine-learning double cross-validation algorithm for variable selection called MUVR,^38^ from the “MUVR” R-package. Changes (Δ post-pre) metabolites and Δ gut microbiota were modeled as independent variables and treatment (rye vs wheat) as the dependent variable. For integrative analysis combining gut microbiota composition and metabolomic changes, we used multiple factor analysis (MFA), which is a multivariate data analysis method that summarizes and visualizes complex data in which individuals are described by several sets of variables (quantitative and/or qualitative) structured into groups. MFA was conducted with “FactoMineR” R-package.^39^ The variables included in the analysis were structured into the following groups: sex, diet, clinical variables at baseline (age, BMI, triglycerides, glucose, cholesterol, and CRP), Three Factor Eating Questionnaire (cognitive restraint, uncontrolled eating, and emotional eating), and Δ metabolites (variables = 37) and Δ gut microbiota (variables = 16) selected with the MUVR model.

Spearman correlations were conducted between Δ gut microbiota (genus level) and Δ metabolites. Furthermore, associations between changes in selected metabolite biomarkers, gut bacteria, and changes in cardiometabolic risk factors were assessed using age-, baseline level-, and sex-adjusted linear mixed models, with random intercepts for the participants. This analysis was further adjusted by changes in the plasma total alkylresorcinol levels.

All statistical analyses were performed using R version 4.2.3 (R Foundation, Austria).

## Results

### Baseline Characteristics in the RyeWeight Study

After excluding 35 participants who dropped out, 207 participants completed the 12-week intervention, 108 for rye, and 99 for refined wheat intervention. Baseline characteristics of the 207 participants can be found in Supplementary Table 1. At baseline, the intervention groups did not differ in terms of anthropometric measures or metabolic characteristics. Sex and age distributions were homogeneous between the groups, but body fat was higher for participants in the wheat group (*p* < 0.05).

### Effect of the Intervention on Cardiometabolic Health

As previously reported by Iversen et al.,^26^ greater reductions in body weight, body fat, BMI, and waist circumference were observed among participants in the rye group compared to the refined wheat group (all *p* < 0.05). In addition, plasma CRP concentrations were lower in the rye group vs the wheat group after 12 weeks (*p* < 0.05). The intervention had no effects on the other clinical markers measured as secondary outcomes of the study (namely, systolic blood pressure, glucose, insulin, HDL cholesterol, and triglycerides) at 12 weeks (Supplementary Table 2).

### Effect of the Intervention in Plasma-Targeted Metabolomics

Several plasma metabolites were significantly increased due to the rye intervention compared to wheat (FDR-adjusted *p*-value <0.05, Figure 1, Supplementary Figure 1, and Table S2). The benzoxazinoid metabolites 2-hydroxy-*N*-(2-hydroxyphenyl)­acetamide sulfate (2 HHPA-S), *N*-(2-hydroxyphenyl) acetamide sulfate (2 HPA-S), 2,4-dihydroxy-1,4-benzoxazin-3-one sulfate (DIBOA-S), and 2-hydroxy-7-methoxy-1,4-benzoxazin-3-one sulfate (HMBOA-S); the phenolic acids gallic acid 4-sulfate and vanillic acid sulfate; the gut microbial metabolite of tryptophan, indolepropionic acid; and pipecolic betaine and succinylglycine were the most affected metabolites by rye intervention (log_2_ fold change >0.58 and FDR-*p*-adjusted value <0.05). Other metabolites that increased after the rye diet were the betainized metabolite phenylalanine betaine; the microbial metabolites 2,6-dihydroxybenzoic acid (2,6-DHBA) and 2-aminophenol; and the endogenous metabolites glutaryl carnitine and asymmetric dimethylarginine.

**Figure 1:**
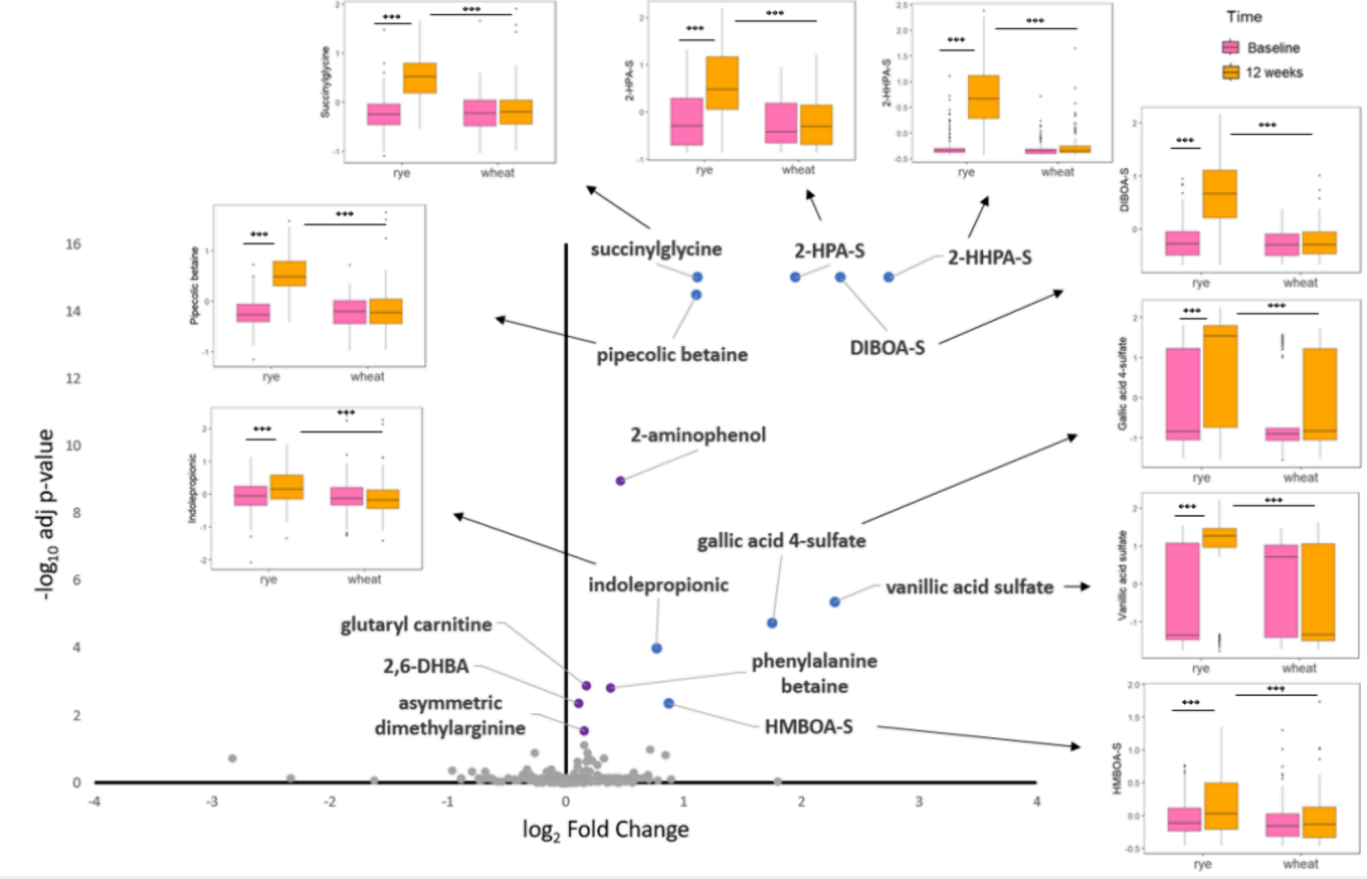
Effects of diet (wheat vs rye) on plasma metabolites (*n*= 207, *k* = 414). According to linear mixed models with random intercepts (defined by participant ID) and modeling diet, time, and its two-way interaction as fixed factors. Metabolites in colors have an FDR-adjusted *p*-value <0.05. Those in blue have a log2fold-change >0.58 while those in purple do not. Boxplots showing the normalized concentration (n.c) distribution for each metabolite with a log2fold change (rye/wheat) >0.58. For metabolites with a log2fold change (rye/wheat) <0.5 (purple dots), see Supplementary Figure 1. 2,6-DHBA, 2,6-dihydroxybenzoic acid; DIBOA-S, 2,4-dihydroxy-1,4-benzoxazin-3-one sulfate; 2-HHPA-S, 2-hydroxy-*N*-(2-hydroxyphenyl)­acetamide sulfate; 2-HPA-S, *N*-(2-hydroxyphenyl)­acetamide sulfate; HMBOA-S, 2-hydroxy-7-methoxy-1,4-benzoxazin-3-one sulfate.

Further multivariate analysis showed similar results with the addition of metabolites derived from gut fermentation of lignans, enterolactone sulfate, and enterolactone glucuronide (Figure 2).

**Figure 2:**
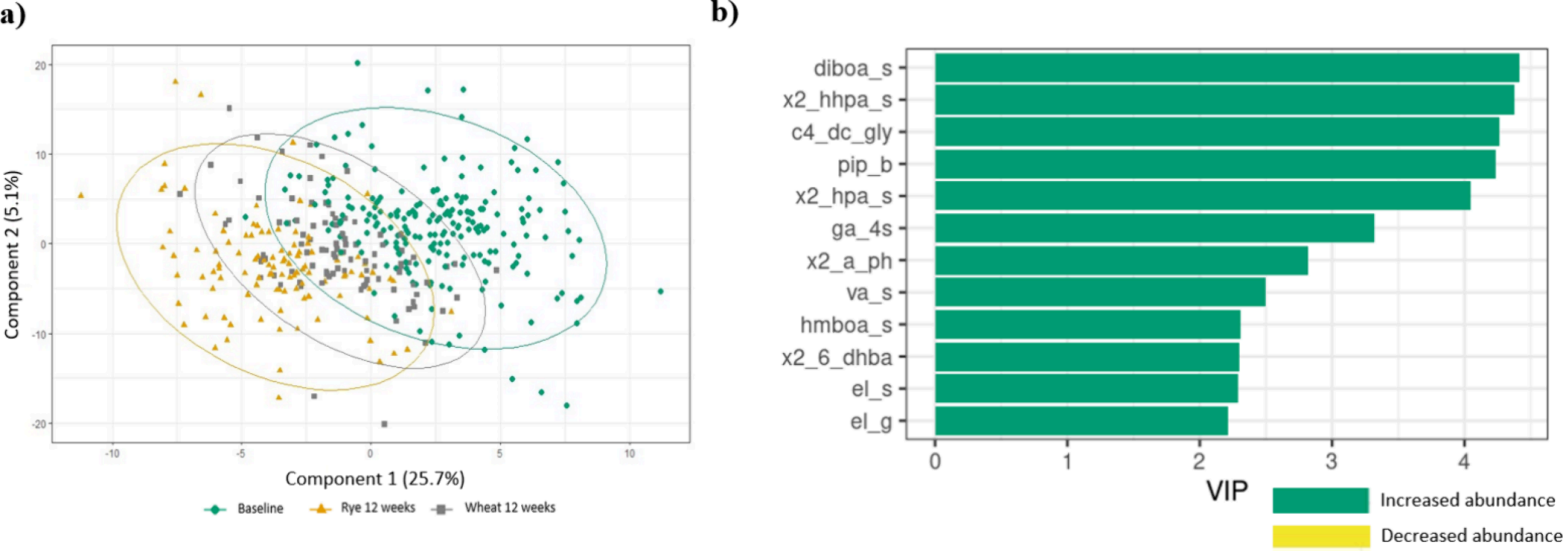
Multilevel partial least-square discriminant analysis (PLS-DA) showing the effect of diet (wheat vs rye) on plasma metabolites (*n*= 207, k = 414). (a) Two-dimensional view showing the distribution of participants at baseline (*n* = 207) and after 12 weeks of the rye (*n* = 108) or refined wheat intervention (*n* = 99) according to the first two components of the PLS-DA model. Ellipses indicate 95% confidence regions for each group. (b) Variable Importance in Projection (VIP) scores showing the most contributory metabolites (VIP >2.0) for diet discrimination in the PLS-DA model. Green represents metabolites increased during the intervention. diboa_s, 2,4-dihydroxy-1,4-benzoxazin-3-one sulfate; x2_hhpa_s, 2-hydroxy-*N*-(2-hydroxyphenyl)­acetamide sulfate; c4_dc_gly, succinylglycine; pip_b, pipecolic betaine; x2_hpa_s, *N*-(2-hydroxyphenyl)­acetamide sulfate; ga_4s, gallic acid 4-sulfate; x2_a_ph, 2-aminophenol; va_s, vanillic acid sulfate; hmboa_s, 2-hydroxy-7-methoxy-1,4-benzoxazin-3-one sulfate; x2_6_dhba 2,6-dihydroxybenzoic acid; el_s, enterolactone sulfate; el_g, enterolactone glucuronide.

### Integrative Analysis Combining Clinical Characteristics, and Targeted Metabolomics and Gut Microbiota Composition Data

We additionally conducted an integrative analysis combining metabolomics data with the gut microbiota composition data. We first used the MUVR-algorithm to identify changes in metabolites and bacteria associated with the dietary intervention. We obtained a selection of 53 variables (MUVR fitness CR = 0.87) (Supplementary Figure 2; the list of selected variables by the model and the median changes in wheat and rye groups is shown in Supplementary Table 3). Robustness of the model for classification was tested using a 100-permutation test, which was of statistical significance *p* < 0.001.

The selection of microbial and metabolomics variables was further included in the MFA model, including the variables sex, baseline clinical variables, and eating behavior assessed by TFEQ, as it has been associated with improved weight loss^40^ and it may have an influence on the outcomes of the high-fiber rye intervention. As observed in Supplementary Figure 3, Dimension 2 of the MFA was the one that allowed for the separation between wheat and rye interventions. Changes in metabolites and gut bacteria were the variable groups contributing the most to describe the effect of diet irrespective of sex or baseline clinical characteristics (Supplementary Figure 3). Variables contributing the most to Dimension 2 are shown in Supplementary Figure 4. Metabolites in Dimension 2 were very similar to those obtained in previous multivariate and univariate analyses, and the bacterial genera *Eubacterium xylanophilum*, *Agathobacter*, *Ruminococcus torques*, *Oscillospiraceae* UCG-003, *Romboutsia*, *Faecalibacterium*, and *Flavonifractor* were highlighted. Agathobacter, *Ruminococcus torques*, and *Oscillospiraceae UCG-003* had previously been associated with this intervention.^31^ However, Romboutsia, *Eubacterium xylanophilum*, *Faecalibacterium*, and *Flavonifractor* were not previously reported to be associated with a wholegrain rye intervention. To describe the relationship between these bacteria and the rye intervention, we correlated the bacterial genus with the metabolites affected by the intervention (Figure 3). Changes in *Eubacterium xylanophilum*, *Agathobacter*, and *Oscillospiraceae UCG-003* were positively correlated with metabolites increased by the wholegrain rye intervention. On the other hand, changes in *Ruminococcus torques*, *Romboutsia*, and *Flavonifractor* were inversely correlated with these metabolites.

**Figure 3:**
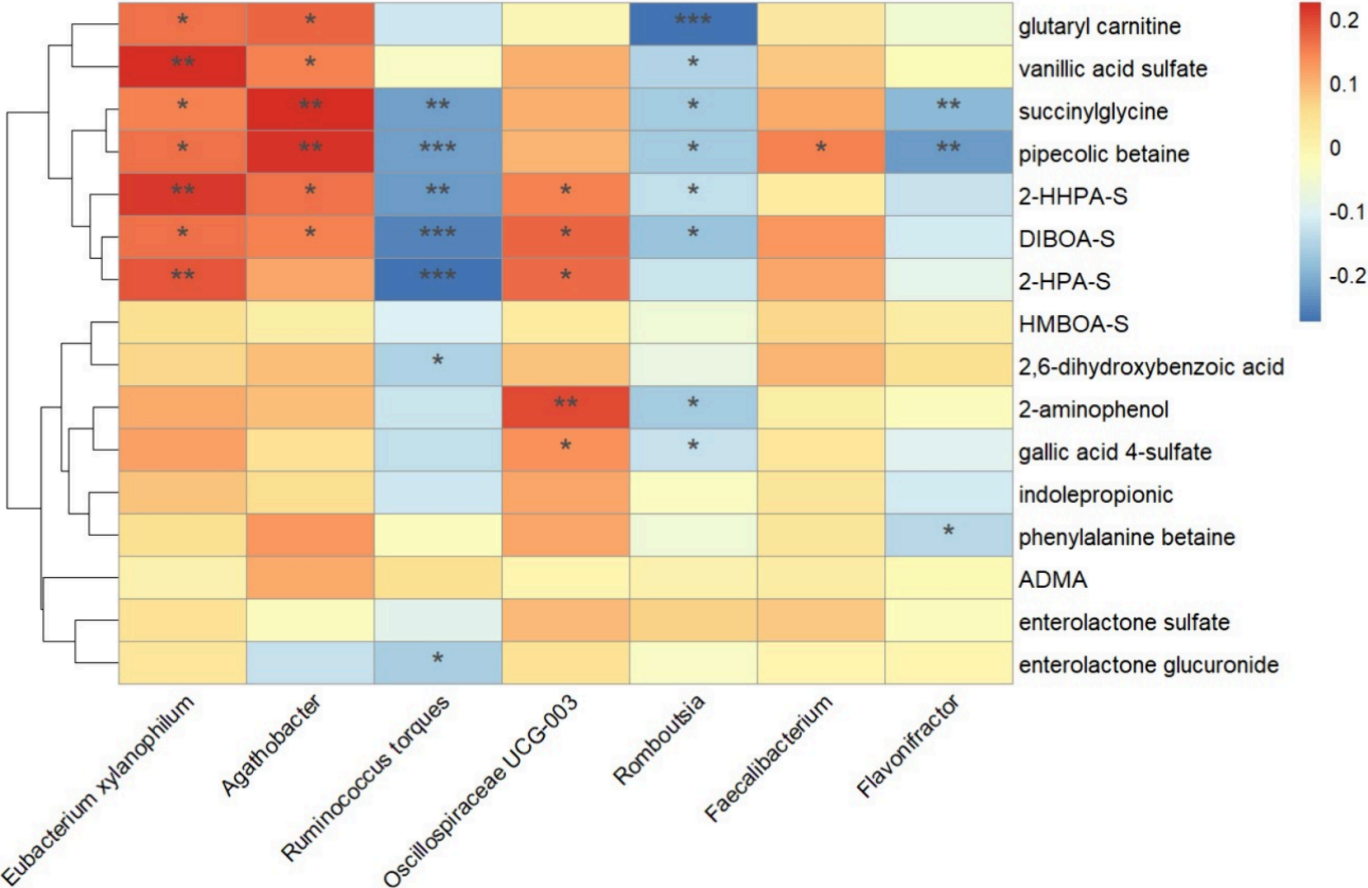
Heatmap showing the correlations between changes in gut microbiota (genus level) and changes in metabolites in all the individuals of the trial (*n* = 207, *k* = 414). Spearman coefficients and *p*-values using Δmetabolites and Δgut microbiota data. *p-value <0.05, **p <0.01, ***p-value <0.001. DIBOA-S, 2,4-dihydroxy-1,4-benzoxazin-3-one sulfate; 2-HHPA-S, 2-hydroxy-*N*-(2-hydroxyphenyl)­acetamide sulfate; 2-HPA-S, *N*-(2-hydroxyphenyl)­acetamide sulfate; HMBOA-S, 2-hydroxy-7-methoxy-1,4-benzoxazin-3-one sulfate.

### Associations between Plasma Metabolites and Cardiometabolic Risk Factors

The association between changes in the previously selected metabolites and changes in body weight and fat mass (primary outcome) and cardiometabolic risk factors (secondary outcomes) was assessed among participants following the high-fiber rye diet (*n* = 108) (Figure 4), as well as among all participants (*n* = 207) and those following the refined wheat diet (*n* = 99) (Supplementary Figure 5). Among participants following the high-fiber rye diet, changes in weight, fat mass, and BMI were inversely associated with changes in 2-aminophenol, DIBOA-S, 2HHPA-S, succinylglycine, pipecolic betaine, asymmetric dimethylarginine (ADMA), and gallic acid 4-sulfate (Figure 4, panel a). As an attempt to consider differences in compliance and intake levels of the intervention products in the association analysis, we additionally adjusted the model by total plasma alkylresorcinols^41^ (Figure 4, panel b). In this analysis, some of the associations did not remain statistically significant; however, the associations of succinylglycine, ADMA, 2-HHPA-S, and gallic acid 4-sulfate with weight loss, fat mass loss, or decreased BMI did remain. Additionally, other inverse associations such as succinylglycine and pipecolic betaine with LDL cholesterol or glutarylcarnitine with glucose were also observed. MFA Dimension 2, integrating changes in metabolites and gut bacteria, was negatively associated with weight and BMI in the first analysis but lost statistical significance when adjusting by plasma total alkylresorcinols. Probably because Dimension 2 encompasses information relevant to describe the dietary intervention but not necessarily weight loss.

**Figure 4:**
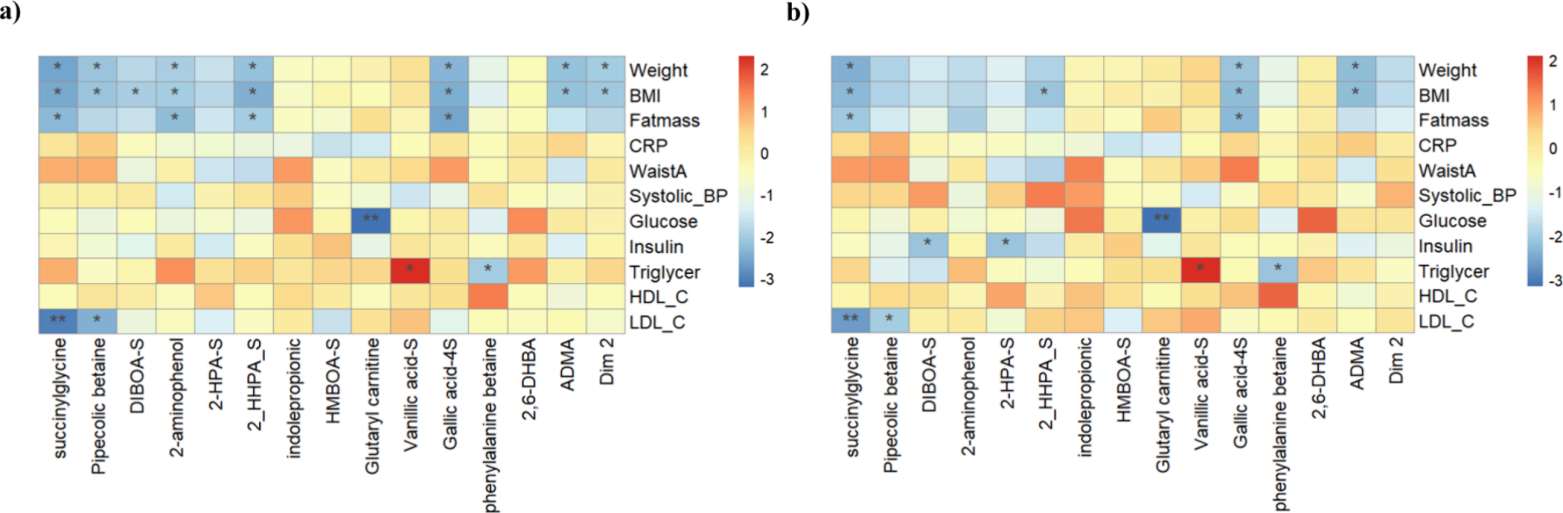
Heatmaps showing the associations between changes in metabolites and changes in cardiometabolic risk factors for participants in the rye group, not adjusted (a) and adjusted by total alkylresorcinols (b). Coefficients and *p*-values using age-, baseline level-, and sex-adjusted linear mixed models using random intercepts for Δmetabolites and Δclinical variables. *p-value <0.05, **p < 0.01, ***p-value <0.001. DIBOA_S, 2,4-dihydroxy-1,4-benzoxazin-3-one sulfate; 2_HPA¬_S, *N*-(2-hydroxyphenyl)­acetamide sulfate; 2_HHPA_S, 2-hydroxy-*N*-(2-hydroxyphenyl)­acetamide sulfate; HMBOA_S, 2-hydroxy-7-methoxy-1,4-benzoxazin-3-one sulfate; 2_6_DHBA, 2,6-dihydroxybenzoic acid; ADMA, asymmetric dimethylarginine; Dim_1,MFA Dimension 1; CRP, C-reactive protein; WaistA, waist circumference; HDL_C, HDL cholesterol; LDL_C, LDL cholesterol.

## Discussion

The present study identified several plasma metabolite biomarkers that were altered with a 12-week high-fiber wholegrain rye diet vs. a refined wheat diet in adults with overweight and obesity. We observed that wholegrain rye increased concentrations of diverse benzoxazinoid metabolites, betainized compounds, some phenolic acids, and microbial metabolites including indolepropionic acid, 2-aminophenol, and enterolactone sulfate and enterolactone glucuronide, as well as diverse endogenous metabolites such as succinylglycine and glutarylcarnitine. We additionally conducted an integrative analysis combining targeted metabolomics, 16S rRNA data, clinical variables, and eating behavior, which showed that the intervention effect was mostly captured by changes in metabolites and gut microbiota independently of anthropometric, clinical, and eating behavior characteristics. Last, the associations between gallic acid-4-sulfate and the sulfonated phenylacetamides, 2-HPA-S and 2-HHPA-S, with reductions in weight, fat mass, BMI, or fasting insulin levels even after adjusting for plasma alkylresorcinols suggest that they may constitute biomarkers of wholegrain rye cardiometabolic effects.

Benzoxazinoids and derived metabolites (DIBOA-S, HMBOA-S, 2 HHPA-S, and 2 HPA-S) were the major family of compounds that were increased after rye intake in this study. Benzoxazinoids are a well-known class of phytochemicals almost exclusively found in wholegrain wheat and rye.^42,43^ Although they have been originally described as part of the defense mechanism of these cereal plants, they have also shown pharmacological and beneficial health properties,^44^ including weight loss and appetite suppression among overweight people.^45^ DIBOA is one of the major benzoxazinoids found in rye bread,^46^ and both HMBOA-S and DIBOA-S as well as the phenylacetamydes HPA-S and HHPA-S have been shown to be important discriminatory metabolites both in plasma and in urine after wholegrain rye foods intake.^47–50^ 2-HPA-S and 2-HHPA-S have not been previously linked with the beneficial metabolic effects of wholegrain rye, and associations between specific benzoxazinoids or derived metabolites with cardiometabolic risk factors have not been yet described, only with risk markers of prostate cancer.^51^ Thus, while some benzoxazinoid-derived metabolites have been associated with weight and fat mass reduction, their underlying mechanisms of action remain to be elucidated. Among the gut bacteria genera described in the integrative MFA, the most positively correlated with phenylacetamyde production was *Eubacterium xylanophilum*, while inverse correlations were described for *Ruminococcus torques*. Of major importance, *E. xylanophilum* genus was increased in individuals with a lower inflammatory diet index^52^ and could be relevant for the global effects of rye intervention.^31^ *E. xylanophilum* is generally considered a strong butyrate producer, specially from flavonoid degradation,^53,54^ and it has been negatively associated with adiposity and visceral adipose tissue.^52,55,56^ Potential pathways of action for reduced adiposity for *E. xylanophilum* could involve its functional capacity of producing butyrate, which has shown to promote thermogenesis in brown adipose tissue by activating lysine specific demythylase,^57^ ultimately leading to reduced body fat and weight loss. No direct link has been reported between *E. xylanophilum* and the specific intake of wholegrain rye. However, a selective enrichment of this bacterium was observed after wheat bran addition,^58^ making it an interesting candidate for future precise microbiome-based interventions with wholegrain cereals. On the other hand, *R. torques* is known to decrease gut barrier integrity,^59,60^ mainly since it is a potent mucus degrader.^61^ In addition, it has been associated with higher blood triglyceride levels and irritable bowel syndrome.^59,61^ Interestingly, *R. torques* has been negatively associated with Mediterranean diet,^62,63^ plant-based foods,^64^ and the previous analyses of this study.^31^ Inverse associations between *R. torques* and wholegrain cereals warrant further research.

The microbial metabolites indolepropionic acid, 2-aminophenol, and 2,6-DHBA were also increased with the high-fiber rye intervention. Indolepropionic acid is a well-known tryptophan metabolite generated by gut microbiome that has been repeatedly associated with the intake of dietary fiber^65,66^ or wholegrain cereals^67^ and linked with several health outcomes.^65,68^ Interestingly, 2,6-DHBA, a phenolic compound of less characterized origin, also increased with rye intervention. Unfortunately, although it is thought to be a microbial metabolite derived from fermentation of lignans, the specific microbial enzymes have not been identified, and the exact dietary sources are yet unknown. Altogether, indolepropionic acid, 2-aminophenol, and 2,6-DHBA appeared to be consistently associated with total dietary fiber intake in a recent study conducted in our group.^66^

The phenolic acid gallic acid 4-sulfate was inversely associated with weight, BMI, and fat mass in the rye group even after adjusting for plasma total alkyresorcinols, well-established biomarkers of wholegrain rye intake.^69^ Gallic acid 4-sulfate is mainly found in vegetables, nuts, and fruits,^70^ but it has also been detected in rye in higher concentrations compared to other cereal grains.^71^ However, it has not been previously postulated as a specific biomarker for rye intake or its metabolic effects. Gallic acid has been shown improvements in metabolic syndrome,^72,73^ probably through effects in energy expenditure, increasing thermogenesis.^74^ Similarly, in line with other studies,^15,75^ pipecolic betaine was inversely associated with LDL cholesterol. However, we have not observed associations with glucose metabolism as reported by others.^76^

Lastly, the endogenous metabolite succinylglycine increased after rye intervention, and curiously, it was inversely associated with main outcomes of the study. Succinylglycine was consistently associated with rye intake in both univariate and multivariate analyses and inversely associated with weight, BMI, and fat mass even when adjusting by total alkylresorcinols. This might show that increments in succinylglycine are a physiological reflection of weight and fat mass loss, since succinylglycine is an intermediate metabolite involved in energy metabolism.^77,78^ In addition, higher succinylglycine could be a way to remove carbons from the Krebs cycle that could result from the excess of acetyl-CoA and NADPH, common to obesity and insulin-resistant states.^79^ Indeed, excretion of acylglycines is usually decreased in obesity and restored after weight loss.^80^ Of major importance, the glycine conjugation pathway is considered a component of the human detoxification system, and the increase in plasma succinylglycine could respond to a higher glycine availability at the liver.^78,80^ Strikingly, the arginine analogue ADMA remained constant during rye intervention but decreased for those in the wheat arm (Supplementary Figure 1). In addition, ADMA appeared to be inversely associated with weight and BMI. Elevated levels of ADMA have been shown to inhibit nitrogen oxide synthesis and therefore impair endothelial function. Indeed, higher plasma ADMA levels have been detected in people with hypercholesterolemia, atherosclerosis, and other cardiometabolic diseases.^81^ The reasons why the reduction in ADMA levels was only significant for those participants in the wheat intervention are unknown but could be related to the effects of the hypocaloric diet. It is necessary to replicate and validate these results in further studies of other populations.

One of the strengths of this study is the use of a high-throughput, comprehensive targeted metabolomics method able to capture a wide spectrum of diet-related and endogenous metabolites. In addition, the study design allowed the assessment of the specific contribution of high-fiber rye to the blood metabolome after a 2-week run-in period with refined wheat. The intervention compliance rate was high as assessed by personal diaries and also included the measurement of validated rye intake biomarkers, such as alkylresorcinol levels.^41^ On the other hand, the run-in period with refined wheat could explain why the metabolites significantly altered during the intervention were only increased in the wholegrain rye group. Moreover, we observed no associations between the metabolites significantly altered by the intervention and reductions in CRP levels, which was one of the interesting findings of the trial. Since the study was designed as a weight loss study, it cannot be excluded that any changes in metabolic risk factors may be confounded by weight loss. In addition, the unavailability of data from the study 6-week time point should be noted. Although this prevents the inclusion of intermediate outcomes, all available samples from participants who completed the study were analyzed at baseline and 12 weeks. More studies are needed to provide external validation for these metabolites as biomarkers of rye intake or as biomarkers of the cardiometabolic effects attributable to rye intake.

In conclusion, the alterations of plasma metabolome induced by a 12-week consumption of high-fiber wholegrain rye intervention included increased levels of benzoxazinoids (DIBOA-S), phenylacetamides, gut microbial metabolites (indolepropionic acid, 2-aminophenol), betainized compounds (pipecolic-betaine), and phenolic acids (2,6-DHBA, gallic acid-4-S). Moreover, changes in gut microbiota (increased*Eubacterium xylanophylum* and *Agathobacter* and decreased *Ruminococcus torques* and *Romboutsia*) and plasma metabolites were sufficient to describe the effects of the intervention disregarding clinical and biochemical characteristics. Phenylacetamide metabolites (2-HPA-S and 2-HHPA-S), gallic acid 4-sulfate, and succinylglycine may constitute biomarkers of wholegrain rye cardiometabolic effects.

## Supplementary Material





## Data Availability

Data is available from the corresponding author upon reasonable request. As the data contains sensitive information the data will not be made publicly available.

## Notes

(Supplementary Table 1) Baseline characteristics of the participants enrolled in the RyeWeight study; (Supplementary Table 2) influence of diet (wheat vs rye) on changes in cardiometabolic risk factors in the RyeWeight study; (Supplementary Table 3) variable importance rank of the metabolites and bacteria associated with dietary intervention by MUVR model; (Supplementary Figure 1) box plots showing distributions of normalized concentrations (n.c) for metabolites with an FDR-adjusted *p*-value <0.05 and log2fold-change <0.58; (Supplementary Figure 2) area under the receiver operating characteristic curves for MUVR model; (Supplementary Figure 3) multiple factor analysis (MFA) for individuals’ separation according to changes (Δ) in plasma metabolomics and gut microbiota composition, baseline clinical variables, and eating behavior assessed by Three Factor Eating Questionnaire (TFEQ); (Supplementary Figure 4) contribution of the first 20 quantitative variables to MFA Dimension 2; (Supplementary Figure 5) heatmaps showing the associations between changes in metabolites and changes in cardiometabolic risk factors for all participants (*n* = 207) (panel a) and for participants in the wheat group (*n* = 99) (panel b) (PDF)
(Table S1) metabolites included in the method and their quantifiers; and (Table S2) summary of the whole plasma metabolome data set assessed using linear mixed models (XLSX)
