# Supplementary material for: Metabolite Biomarkers Linking a High-Fiber Rye Intervention with Cardiometabolic Risk Factors: The RyeWeight Study
Source: J Agric Food Chem. 2025 Aug 21;73(35):21869–79. doi: 10.1021/acs.jafc.5c01415 (PMC12412176; doi:10.1021/acs.jafc.5c01415)
Supplement: Supplementary file 1 [file jf5c01415_si_001.pdf]

SUPPLEMENTARY MATERIAL

Metabolite biomarkers linking a high-fibre rye intervention with cardiometabolic risk factors: the RyeWeight study

Andrea Unión Caballero1,2#, Tomás Meroño1,2#, Sebastian Åberg3, Elise Nordin3, Johan Dicksved4, Alex Sánchez-Pla2,5, Marta Cubedo2,5, Francisco Carmona-Pontaque2,5, Kia Nøhr Iversen3, Miriam Martínez-Huélamo1,2, Anna Guadall1,2, Rikard Landberg3, Cristina Andrés-Lacueva1,2*

Affiliations

1. *Biomarkers and Nutrimetabolomics Laboratory, Department de Nutrició, Ciències de l'Alimentació i Gastronomia, Institut de Recerca en Nutrició i Seguretat Alimentària (INSA-UB), Facultat de Farmàcia i Ciències de l'Alimentació, Universitat de Barcelona, 08028 Barcelona, Spain.*
2. *Centro de Investigación Biomédica en Red de Fragilidad y Envejecimiento Saludable (CIBERFES), Instituto de Salud Carlos III, Madrid, 28029, Spain*
3. *Department of Life Sciences, Division of Food and Nutrition Science, Chalmers University of Technology, SE-412 96 Gothenburg, Sweden.*
4. *Department of Applied Animal Science and Welfare; Nutrition and Management, SE-750 07 Uppsala, Sweden*
5. *Department of Genetics, Microbiology and Statistics, University of Barcelona, 08028, Barcelona, Spain.*

# Equally contributing authors

**Corresponding authors*

Supplementary Table 1. Baseline characteristics of the participants enrolled in the RyeWeight study

	Rye<br>n= 108	Wheat<br>n= 99
Sex (%females)	58.7	62.8
Age (years)	58 (49-65)	59 (50-65)
Body weight (kg)	87.6 (77.5-96.1)	86 (80.3-97.5)
BMI (kg/m2)	29.4 (27.8-31.4)	30.1 (28.3-32.4)
Body fat (%)	39.2 (33.9-44.1)	41.9 (36.4-46.2) *
Waist (cm)	102.8 ± 9.3	102.3 ± 9.7
Sagittal diameter (cm)	22.4 ± 2.77	22.0 ± 2.67
Systolic BP (mmHg)	126 (117.1-134.5)	123 (112.4-132)
Glucose (mmol/L)	5.5 (5.2-5.8)	5.5 (5.2-5.9)
Insulin (mIU/L)	8.6 (6.6-12.3)	10.1 (7.1-13.1)
Triglycerides (mmol/L)	1.04 (0.85-1.28)	1.12 (0.88-1.35)
HDL cholesterol (mmol/L)	1.4 (1.1-1.6)	1.4 (1.2-1.6)
CRP (mg/L)	1.5 (0.7-3.2)	1.5 (0.89-2.5)

BMI, body mass index; BP, blood pressure; CRP, C-reactive protein. Values are presented as mean ± S.D. or median (Q1-Q3), according to data following a normal or skewed distribution, respectively. *p <0.05

Supplementary Table 2. Influence of diet (wheat *vs.* rye) on changes in cardiometabolic risk factors in the RyeWeight study.

	**Rye (n=108)**		**Wheat (n=99)**		**P for Diet**	**P for time**	**P for Diet x time**
	**Week 0**	**Week 12**	**Week 0**	**Week 12**			
**Body weight (kg)**	87.8 (77.9-96.6)	85.5 (75.1-93.3)	86.2 (81.4-97.5)	84.8 (79.7-95.9)	0.73	<0.001	**0.005**
**Body fat (%)**	39.2 (34.1-44.1)	37.2 (31.4-42.7)	41.9 (36.5-46.2)	40.6 (34.9-45.4)	0.13	<0.001	**0.006**
**BMI (kg/m2)**	29.4 (27.8-31.4)	28.4 (26.9-30.9)	30.1 (28.3-32.4)	29.6 (27.7-31.7)	0.07	<0.001	**0.005**
**Waist (cm)**	102.8 ± 9.3	99.2 ± 10.2	102.3 ± 9.7	100.5 ± 9.7	0.62	<0.001	**0.007**
**Systolic BP (mmHg)**	126 (117.3-134.5)	124.5 (114.4-137.4)	123 (112.5-131.5)	123 (116-130.9)	0.13	0.58	0.99
**Glucose (mmol/L)**	5.5 (5.2-5.7)	5.4 (5.2-5.7)	5.5 (5.2-5.7)	5.4 (5.2-5.8)	0.26	0.002	0.47
**Insulin (mIU/L)**	8.6 (6.6-12.2)	8.3 (5.9-10.8)	10.1 (7.1-13)	9.3 (6.6-13.1)	0.26	0.005	0.57
**HDL cholesterol (mmol/L)**	1.4 (1.1-1.6)	1.4 (1.2-1.6)	1.4 (1.2-1.6)	1.5 (1.3-1.7)	0.09	0.014	0.14
**Triglycerides (mmol/L)**	1.04 (0.85-1.27)	1.11 (0.92-1.43)	1.12 (0.88-1.34)	1.11 (0.86-1.39)	0.49	0.018	0.076
**CRP (mg/L)**	1.5 (0.73-3.2)	1.1 (0.67-2)	1.5 (0.89-2.5)	1.7 (0.8-2.7)	0.91	0.005	**0.003**

BP, blood pressure; CRP, C-reactive protein. Values are presented as mean ± S.D. or median (Q1-Q3), according to data following a normal or skewed distribution, respectively. P-values from linear mixed models including individual-specific random effects, and diet (rye *vs.* wheat) and time (week 0 *vs.* week 12) as main effects, with its two-way interaction. Data are presented only for participants who completed the whole intervention (per protocol analysis). Intention to treat analysis were not different and are presented in Iversen *et al.* (26).

Supplementary Table 3. Variable importance rank of the metabolites and bacteria associated with dietary intervention by MUVR model (lower rank means higher importance). Median Δ (post-pre) values for these bacteria and metabolites among rye and wheat groups are also shown

Name	Rank	Median Δ Rye	Median Δ Wheat
diboa_s	2.295.556	0.91	0.03
x2_hhpa_s	2.320.000	0.95	0.01
pip_b	2.635.556	0.78	0.00
c4_dc_gly	2.902.222	0.75	0.04
x2_hpa_s	4.985.556	0.79	0.04
x2_a_ph	6.393.333	0.37	-0.01
va_s	7.381.111	0.64	-0.02
indolepropionic	20.284.444	0.32	-0.06
[Ruminococcus] torques group	25.054.444	-0.60	-0.12
ga_4s	27.700.000	0.75	0.08
c5_0_dc_car	36.056.667	0.10	-0.02
phe_b	38.511.111	0.18	-0.02
Romboutsia	44.473.333	-0.23	0.22
lpe18_1_i1	58.933.333	0.28	-0.12
[Eubacterium] xylanophilum group	66.208.889	0.37	-0.07
Bifidobacterium	73.037.778	0.88	0.53
Lachnospiraceae AC2044 group	86.818.889	0.54	0.24
x3_hphpa	90.188.889	0.36	-0.09
x2_6_dhba	99.096.667	0.28	0.07
tyra_s	102.448.889	0.06	-0.01
Colidextribacter	153.977.778	-0.11	0.04
[Eubacterium] siraeum group	154.894.444	-0.41	-0.10
dimethylglycine	164.548.889	0.05	-0.06
methionine	164.776.667	0.02	0.04
hmboa_s	166.862.222	0.12	-0.01
Flavonifractor	167.863.333	-0.73	-0.26
cml	174.834.444	0.04	-0.09
n_acetylglycine	177.805.556	0.08	-0.02
x4_hpla	185.720.000	-0.06	0.02
NK4A214 group	199.814.444	0.32	0.10
UCG-003	200.614.444	0.53	0.25
el_s	202.443.333	0.26	0.11
Agathobacter	202.464.444	0.34	0.08
dhfa_s	208.947.778	0.01	0.00
x4_pyridoxic	216.217.778	0.04	-0.02
serotonin	223.072.222	0.07	-0.03
x3_4_dhba	223.817.778	0.10	-0.05
in_s	225.653.333	0.02	0.13

Megamonas	237.161.111	0.07	-0.02
Blautia	239.026.667	-0.21	-0.04
dhfa	239.417.778	0.04	0.01
Anaerostipes	240.932.222	0.33	0.09
asymmetric_dimethylarginine	242.086.667	0.00	-0.12
x3_hb_ald_x4_hb_ald	242.920.000	-0.01	0.04
x4_hha	250.283.333	0.18	0.00
[Eubacterium] ventriosum group	251.113.333	-0.70	-0.34
c6_0_dc_car	254.791.111	0.01	-0.09
Faecalibacterium	259.702.222	0.12	-0.02
c8_0_dc_car	261.973.333	-0.01	-0.13
x3_mx	266.086.667	0.14	-0.18
x3_5_dhba	266.871.111	0.07	-0.12
mg16_0	281.535.556	0.07	0.00
dha	282.384.444	-0.01	0.20

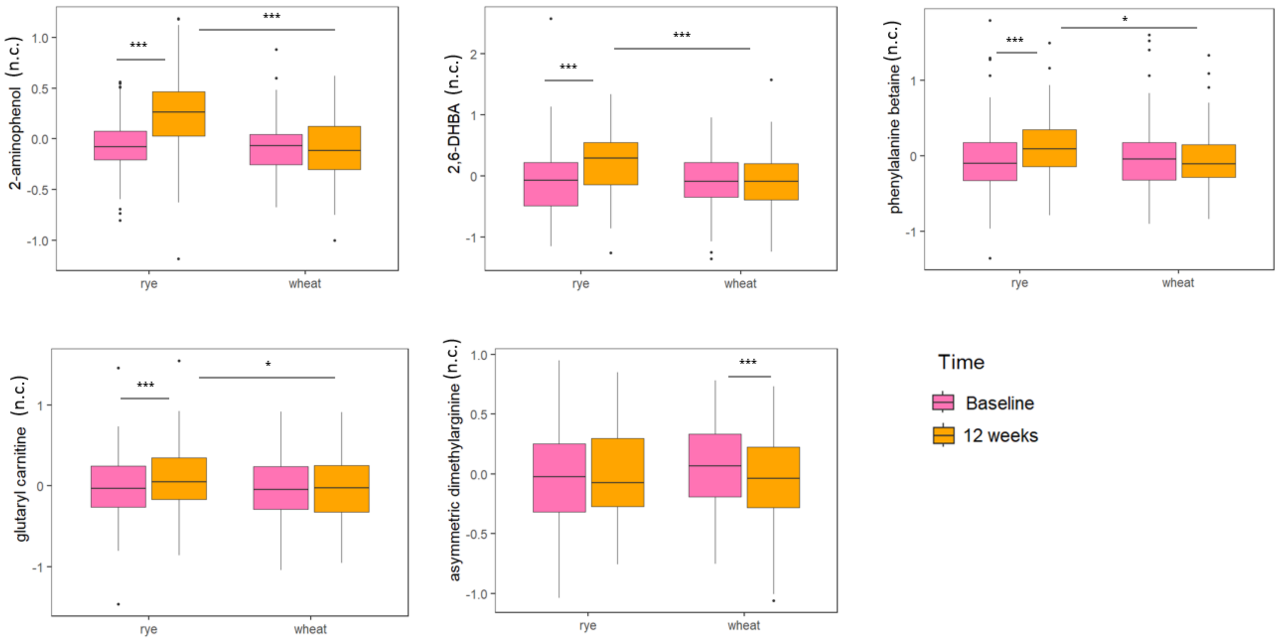

Supplementary Figure 1. **Box plots showing distributions of normalized concentrations (n.c) for metabolites with an FDR-adjusted p-value <0.05 and log2fold-change<0.58 according to linear mixed models with random intercepts (defined by participant ID) (n=207, k=414).**

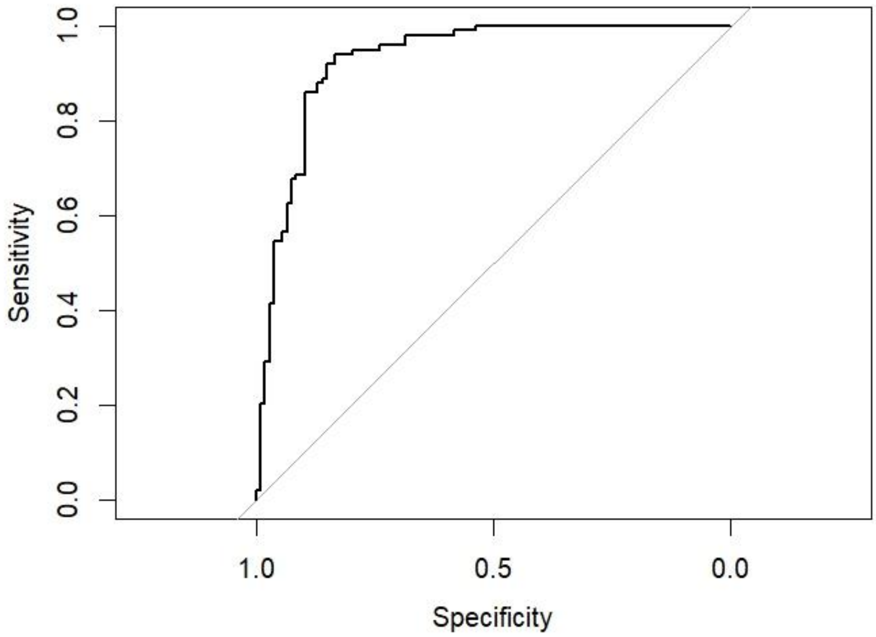

Supplementary Figure 2. Area under the receiver operating characteristic curves for MUVR model.

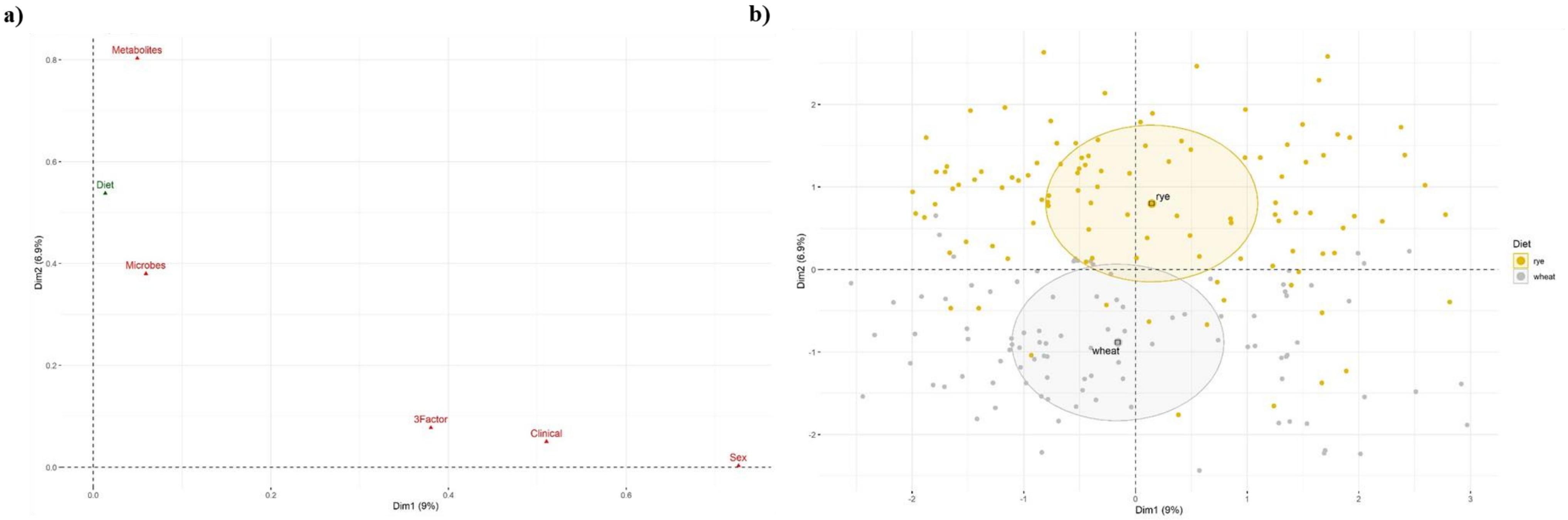

Supplementary Figure 3. Multiple factor analysis (MFA) for individuals' separation according to changes (Δ) in plasma metabolomics and gut microbiota composition, baseline clinical variables and eating behaviour assessed by Three Factor Eating Questionnaire (TFEQ) (n=207, k=414). a) Variable groups contributing to Dimensions 1 and 2. **b)** Individuals' separation according to MFA analysis in the first two dimensions of the analysis. Diet, intervention (rye *vs.* wheat); Metabolites, Δ metabolites from MUVR model; Microbes, Δ gut microbiota from MUVR model; 3 Factor eating behaviour variables assessed by TFEQ; Clinical, baseline clinical variables (age, BMI, triglycerides, glucose, cholesterol and CRP).

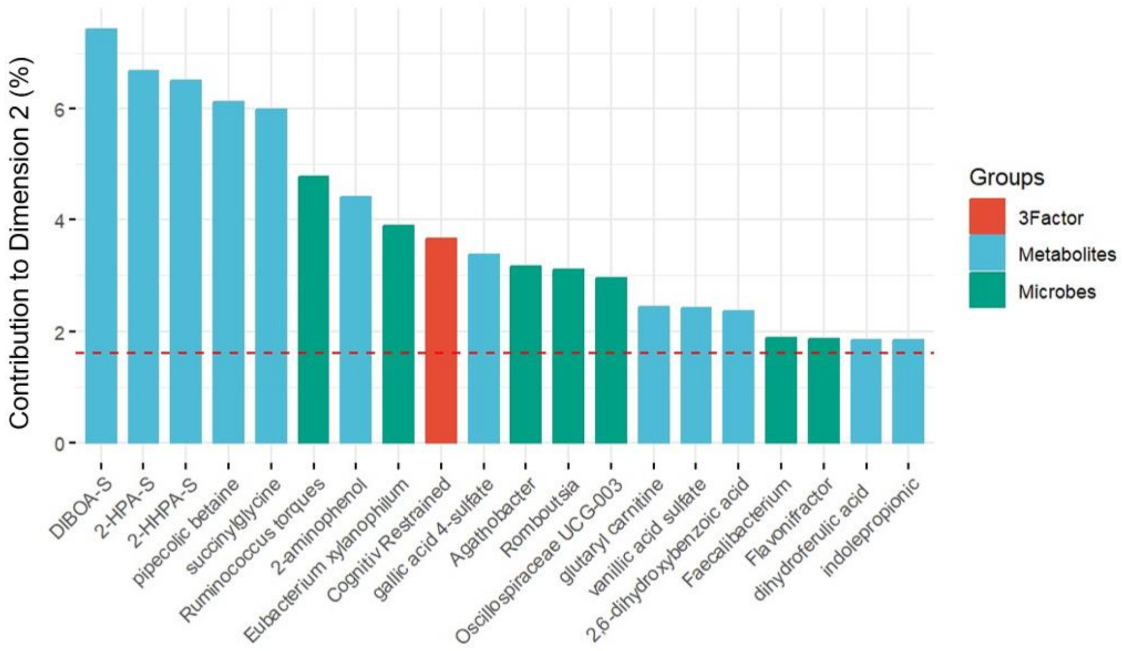

Supplementary Figure 4. Contribution of the first 20 quantitative variables to MFA Dimension 2.

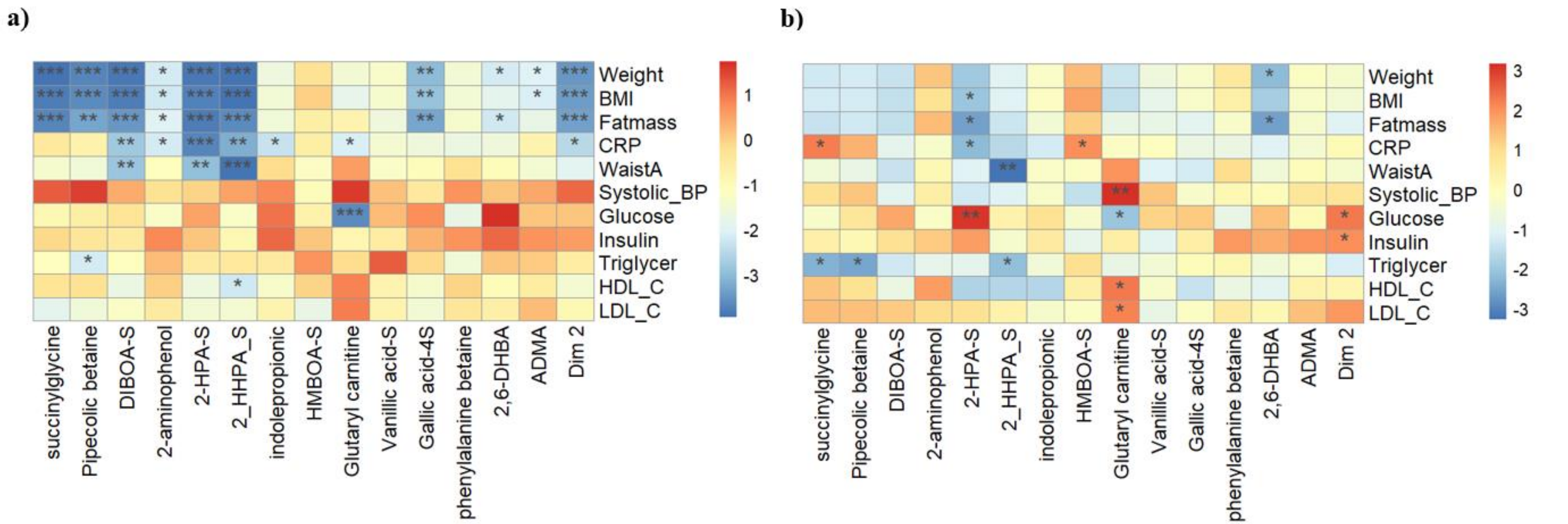

Supplementary Figure 5. Heatmaps showing the associations between changes in metabolites and changes in cardiometabolic risk factors for all participants (n=207) (panel a) and for participants in the wheat group (n=99) (panel b). Coefficients and p-values using age-, baseline levels- and sex- adjusted linear mixed models for Δ metabolites and Δ clinical variables with random intercepts. * p-value<0.05, ** <0.01, *** p-value <0.001. DIBOA_S, 2,4-dihydroxy-1,4-benzoxazin-3-one sulfate; 2_HPA¬_S, N-(2-hydroxyphenyl)acetamide sulfate; 2_HHPA_S, 2-hydroxy-N-(2-hydroxyphenyl)acetamide sulfate; HMBOA_S, 2-hydroxy-7-methoxy-1,4-benzoxazin-3-one sulfate; 2_6_DHBA, 2,6-dihydroxybenzoic acid; ADMA, asymmetric dimethylarginine; Dim_1,MFA Dimension 1; CRP, C-reactive protein; WaistA, waist circumference; HDL_C, HDL cholesterol; LDL_C, LDL cholesterol.
